# Observational articles: a tool to reconstruct ecological history based on chronicling unusual events

**DOI:** 10.12688/f1000research.2-168.v1

**Published:** 2013-08-09

**Authors:** Ferdinando Boero

**Affiliations:** 1 Universita' del Salento, 73100 Lecce, Italy; 2 National Research Council, Institute of Marine Sciences, Genoa, 16149, Italy

## Abstract

Natural history is based on observations, whereas modern ecology is mostly based on experiments aimed at testing hypotheses, either in the field or in a computer. Furthermore, experiments often reveal generalities that are taken as norms. Ecology, however, is a historical discipline and history is driven by both regularities (deriving from norms) and irregularities, or contingencies, which occur when norms are broken. If only norms occured, there would be no history. The current disregard for the importance of contingencies and anecdotes is preventing us from understanding ecological history. We need rules and norms, but we also need records about apparently irrelevant things that, in non-linear systems like ecological ones, might become the drivers of change and, thus, the determinants of history. The same arguments also hold in the field of evolutionary biology, with natural selection being the ecological driver of evolutionary change. It is important that scientists are able to publish potentially important observations, particularly those that are unrelated to their current projects that have no sufficient grounds to be framed into a classical eco-evolutionary paper, and could feasibly impact on the history of the systems in which they occurred. A report on any deviation from the norm would be welcome, from the disappearance of species to their sudden appearance in great quantities. Any event that an “expert eye” (i.e. the eye of a naturalist) might judge as potentially important is worth being reported.

## Introduction

Modern ecology started when Elton (1927) ^1^ published his masterpiece “ *Animal Ecology*” and wrote:

*"Ecology is a new name for a very old subject. It simply means scientific natural history...the words ‘natural history’ bring up a rather clear vision of parties of naturalists going forth on excursion, prepared to swoop down on any rarity that will serve to swell the local list of species. It is a fact that natural history has fallen into disrepute..."*

At the onset of ecology, it was felt that the new science should be better formulized, with the identification of “rules” that were more than the simple “story telling” that was so deprecated by Elton (1927) ^1^. And rightly so. But we have since started to perceive that these “rules” are rather flexible. As a matter of fact, this flexibility had already been perceived before Elton by the father of ecology, Charles Robert Darwin, who, in the *Origin of Species* (1859) ^2^ wrote:

*"Throw up a handful of feathers, and all must fall to the ground according to definite laws. But how simple is this problem compared to the action and reaction of the innumerable plants and animals which have determined, in the course of centuries, the proportional numbers and kinds of trees now growing on the old Indian ruins"*.

Eighty-five years after Elton, the President of the American Society of Naturalists, Robert Ricklefs (2012) ^3^, felt the need to stress the importance of natural history as a potential source of insight in ecology and evolutionary biology, and I took the chance to signal his article to the readers of F1000Prime ^4^. I have also advocated the importance of natural history in previous publications (e.g. Boero and Bondorff 2007 ^5^, Boero *et al.* 2008 ^6^ and references therein).

There are no equations describing the succession of phases leading to a community and there is no unified model for this process. Connell and Slatyer (1977) ^7^, for instance, identified three models, and each time one proves valid, the universality of the others is falsified (if we want to use Popperian logic in ecology) ^8^. From an epistemological point of view, the three models (facilitation, inhibition, tolerance) exist but none of them are universal. Furthermore, in most cases the communities evolve through a blend of models, with a host of *ad hoc* explanations. The same is true in evolutionary biology, with neither gradual nor punctuated models being universal norms.

Ecology, just like evolutionary biology, is a historical discipline. What we see today is the result of a series of events that lead to other events. Sometimes, such as with gradual evolution, the pattern of events follows a gradual trend that might even be predictable but, at other times, there can be special events or contingencies that produce great changes in an abrupt fashion. The great changes, today, are called regime shifts, and they occur when tipping points are reached. Chaos theory, which describes non linear systems, teaches us that apparently negligible events can have a disproportionate bearing on the functioning of a given system. The negligibility of these events is due to the fact that, often, when they occur, their impact is very low. But, sometimes, the impact can be very big.

When a regime shift, or an otherwise unexpected change, occurs we are surprised and it is difficult to determine what the drivers of change might have been. Sometimes, we might detect the triggering event *a posteriori*, but we often cannot, simply because we do not have the information available.

Simply describing what we see is not considered very scientific nowadays and ‘descriptive science’ has become a derogatory term. We must have a hypothesis to test, or better, a controlled experiment that can be performed to identify ecological rules and laws. However, if “ecological rules” were followed by all systems, unexpected things would not happen, which is evidently not the case. Deviations from rules are the main determinants of history, but we cannot test something that is unexpected. As such, our quest to identify rules and regularities could be preventing us from understanding the history of these systems. Paradoxically, we aim at understanding historical systems while using ahistorical approaches! We need a means of reporting these contingencies so that we can better understand the historical trajectory of ecological systems.

The real world out there is like a gigantic jigsaw puzzle where many pieces are missing. Some pieces can be determined through planned projects that seek to test hypotheses, but if another piece is unexpectedly observed in the process, it might as well be collected rather than discarded.

## Publishing observation articles

Long term series are the only way to follow ecological histories but, unfortunately, these are becoming increasingly rare since they do not serve to test specific hypotheses, even though they are crucial for understanding the history of ecological systems. If we are talking about global change, for instance, we must be able to compare the situation of today with that of yesterday, and identify when the main changes occurred, while linking them to possible drivers. But current research trends prevent us from recording this information since we have stopped storing observations in scientific publications. Sometimes, unusual events attract the attention of the media, such as jellyfish blooms, but are disregarded by scientists who are not directly interested in them.

The under-appreciated importance of natural history can also lead to the under-reporting of rare (but important) events, which in turn may lead to ecological patterns not being accepted by others in the research community. For instance, Condon *et al.* (2012) ^9^ questioned shifts from fish-dominated to jellyfish-dominated oceans, considering the phenomenon “unsubstantiated” due to a lack of a substantial number of accounts published in the scientific literature, with reports of jellyfish bloom sightings more prevalent in the popular press. However, the problem is that a jellyfish bloom *per se*, does not have any interest for a scientific journal. It is considered just an observation, an anecdote with no hypothesis to test. As such, even if the frequency of jellyfish bloom sightings increases, the proof of this will be lacking in the scientific record. This is explicitly stated by Riisgård *et al.* (2012) ^10^:

*"The important former commercial fishing of plaice, cod, eel and flounder has been replaced by a countless number of commercially ‘useless’ jellyfish, but no monitoring data have systematically been collected in order to document and to understand the undesirable change within the ecosystem substituting fish with jellyfish as top-predators. Obviously, the basic problem is eutrophication, but knowledge about when, why and how jellyfish became a major pelagic group of key organisms remains largely unknown"*.

We need to be able to collect apparently irrelevant information that might result, when assembled, in a goldmine of facts, which could help us to understand why certain ecological changes occur. Such observations should be written up and submitted to journals that accept submissions based on single observations, including *Herpetology Notes* (herpetology), *Marine Biodiversity Records* (marine biology) and *F1000Research* (all fields). All of these observations, once pooled together, might help build projects with hypotheses to test, whilst arranging observations into a chronological chain of events may help to determine how past contingencies may have led to present systems.

## ‘Scientist science’, that’s what we want

Citizen science, where scientists ask for the help of citizens to gather information, is a popular approach to collecting data in ecology. It is increasingly used as a means to gather information about species over a wide geographical area that scientists would be unable to cover by themselves. For instance, I have carried out a citizen science experiment on jellyfish that began in 2009 and have learnt much from it ^11, 12^. The database of this experiment is a collection of anecdotes but, once charted, provides a clear picture.

Despite the advantages of citizen science, sometimes an “expert eye” can see a potentially important event that might pass unnoticed if observed by lay people. Ecologists and environmental scientists go into the field to carry out their projects and will naturally focus their attention on the variables that are relevant to their projects. However, they often see many other things that may be irrelevant to the fulfilment of their objectives, but that could be of ecological interest and it would be useful if this information was captured and disseminated. For instance, I may be on a cruise, and see a massive bloom of a species (e.g. a jellyfish, or a red tide); I may then go diving, and see that all the sponges are dead.

Perhaps even negative observations can be worthy of publication. It may be the case that I used to see lots of specimens of species X when I started my career, but have not seen any in the last decade. Extinction is a negative result and one that is of ecological interest. If a notable vertebrate species such as a dolphin or a fish becomes extinct, then the International Union for Conservation of Nature may express some concern, but inconspicuous invertebrates often do not make these lists. Most biodiversity is made of inconspicuous stuff. Even a monstrosity might be worth reporting. Monstrous specimens might just be one-off freaks, but then if more and more such individuals are sighted at some specific location, or over a vast area, this could be an indicator that there is some problem in the environment.

In summary, I urge field scientists to take note of anything they see that is unusual and worth recording in their expert eyes. Take some pictures, write a report and submit it to a journal that accepts observation articles. Each observation that is published can be cited; it does not take much of your time and can enhance your CV. We need natural history, we need scientists who can use the most fantastic instrument in the known universe - the human eye. Darwin is our beacon. He collected an enormous number of apparently irrelevant observations during his voyage with the Beagle, continued to observe at home, and treasured the anecdotes that his correspondents sent him. With that mass of little observations, he assembled the theories of both evolution and ecology in one shot.
