# Supplementary material for: Observational articles: a tool to reconstruct ecological history based on chronicling unusual events
Source: F1000Res. 2013 Aug 9;2:168. [Version 1] doi: 10.12688/f1000research.2-168.v1 (PMC3782345; doi:10.12688/f1000research.2-168.v1)
Supplement: Peer review history

# Peer review: Referee response for version 1

**Published:** 2013-08-27

**Authors:** John Chapman

**Affiliations:** 1 Department of Fisheries and Wildlife, Oregon State University, Corvallis, USA

I think this paper is a good idea, and agree that “unusual” events and anecdotes (particularly when they are brief and to the point) are not published enough. The jellyfish blooms are a great example, but dead zones and massive invertebrate changes are also. The article is well organized and to the point. I think that quantitative and experimental ecologists have been vexed for the last 100 years or so, with the daunting problem of measuring anything in nature on the scale that nature occurs. We only have samples and can only measure a small fraction of interactions over very short periods. Anecdotes and periodic observations over time could help fill out the mosaic of ecological dynamics, but we must also admit that we can never see the whole picture.

I have read this submission. I believe that I have an appropriate level of expertise to confirm that it is of an acceptable scientific standard.

# Peer review: Referee response for version 1

**Published:** 2013-08-14

**Authors:** Joachim Mergeay

**Affiliations:** 1 Instituut voor Natuur- en Bosonderzoek, Brussels, Belgium

Ferdinando Boero's plea for documenting unusual events in 'life sciences' is a really important one, but there are some points of discussion. When we try to understand and predict ecological patterns and events, we use universalities, general rules in a probabilistic sense. Whereas ecologists and evolutionary biologists tend to search for generalities, in order to discover general but still probabilistic rules in the mass of data and variables influencing our dependent variables (all kinds of life-forms), we find it hard to acknowledge that these may be very weak at predicting the fate of individual systems. Ultimately, predictions about individual systems is what policy is interested in. Politicians don't want to hear about projections about the average response of a thousand parallel earth-like systems to climate change, they want us to predict the exact response of this planet and its biota to the human CO _2_-pump. But because we can only "predict" based on past observations - history - we tend to predict the response averaged over a large number of hypothetical systems that behaved according to rules of the past. Due to contingencies, this average predicted response may strongly differ from the actual response of the individual system we're actually interested in. And every time we are proven, a posteriori, wrong in our predictions, policy loses faith in soft - highly probabilistic - sciences like ecology and evolutionary biology. It is therefore tremendously important to be aware of the enormous role of contingencies in the historical system that is our world.

To use an analogy from dispersal ecology: modelling a dispersal kernel is not that difficult, except for the shape and length of the tail; rare dispersal events over long distances (at the tail of the distribution) may have large influences (order-of-magnitude!) on the spread rate of organisms (e.g., Clark *et al.* 2003: Ecology 84:1979-88). So it's not enough to know the average response of organisms, outliers can be equally important. It is therefore also important to publish anecdotal facts, observations that may not be framed in a current hypothesis or are part of a large dataset, or which add to existing data. In the face of global change, the oddball of today may be the norm of tomorrow. Deviations from the norm, as Boero puts it, are just as key to understanding life, ecology and evolution, and how we function on this planet.

As mentioned in the paper, citizen science can be a very powerful tool for this. I disagree, however, that we need less of that and more of similar scientist science. There are currently large observation databases based on what citizens observe on their favourite taxonomic groups. They enter their anecdotal data or complete datasets in repositories such as www.observado.org (worldwide), www.waarneming.nl (the Netherlands), www.observations.be (Belgium), http://data.nbn.org.uk/ (UK), and participate in long-term monitoring schemes and so on. Many other observations and datasets are published through www.GBIF.org and related data-portals such as http://bison.usgs.ornl.gov/. Moreover, the real taxonomic specialists are increasingly being found among citizens rather than among scientists. Partly, that's the result of science funding focusing less on natural history and taxonomy, but it's also the result of a world in which digital data and information is accessible much more than it was a decade (or two) ago. Add this availability of information to rapid interaction with peers from all over the world, including rapid dissemination of documentation and evidence, and easy cross-checks of observations (for example by photographic documentation), and becoming a citizen expert is much easier than a decade or two ago. The role of citizen science, also for documenting anecdotal facts, will only increase. 

1. Overall, this paper made me think about how to best deal with anecdotal data: Is the best way to deposit observational data through publication as a single remarkable fact, in a specialized journal willing to publish such data, or to deposit them in citizen-science like databases? Boero argues that once published in a journal, the data is available. But that doesn't necessarily make these observations and data very accessible.
2. Boero argues that even writing anecdotal (typically low-impact) data-papers is still good for your career. But there are just 24 hours in a day. Many scientists have more than enough data in their drawers to publish, but lack time to publish them. How many zombie manuscripts remain unpublished forever? I have a dozen or so hoping to be published sooner or later, but I won’t bet money on them getting published soon. In the rat race for funding, we need high-impact papers, preferably many. So we make a selection of what to publish first, and we tend to aim for expected impact. Writing a high-impact paper does not require much more time than writing a low-impact paper and since time is limited, we focus on writing the former, and skip the scientifically less impacting descriptive anecdotal papers. It’s a real pity these papers and the data behind them don’t get published, but that’s the reality of how science and funding forces us to work. If there’s a fast way to publish anecdotal data in scientific journals, and which doesn't require a lot of writing effort, I think many would welcome this way of publishing.
3. Next, is there really a shortage of journals wanting to publish anecdotal observations? Since the digital era of publication, the number of journals has sky-rocketed, and authors are continuously spammed with unsolicited requests to write papers for new journal X or Y. This said, it would be good to have dedicated journals for this purpose. At the very least, anecdotal data should be deposited in existing data repositories to make them truly accessible. The GBIF data portal or similar initiatives seems like a good initiative for this.

I have read this submission. I believe that I have an appropriate level of expertise to confirm that it is of an acceptable scientific standard.
